# Single-Cell Transcriptomic Analysis Reveals Early Transcriptional Heterogeneity of Cardiac-Associated Cell Populations During Zebrafish Embryogenesis

**DOI:** 10.3390/biology15100791

**Published:** 2026-05-15

**Authors:** Samer N. Khalaf, Mundher Jabbar Al-Okhedi, Amal Saeed Alayed, Mariam M. Jaddah, Asra’a Adnan Abdul-Jalil

**Affiliations:** 1 Department of Biotechnology, College of Sciences, University of Anbar, Ramadi 31001, Iraq; 2 Department of Laboratories Techniques, College of Health and Medical Technology, University of Al Maarif, Ramadi 31001, Iraq; monther.jabbar@uoa.edu.iq; 3 Ministry of Education, Al-Anbar Education Directorate, Ramadi 31001, Iraq; 4 Department of Biological Sciences, Faculty of Science, King Abdulaziz University, P.O. Box 80200, Jeddah 21589, Saudi Arabia; aaied@kau.edu.sa; 5 Department of Clinical Laboratory Sciences, College of Applied Medical Sciences, Taibah University, Madinah 42353, Saudi Arabia; mjidah@taibahu.edu.sa; 6 College of Pharmacy, University of Anbar, Ramadi 31001, Iraq; sc.dr_asraa2017@uoanbar.edu.iq

**Keywords:** zebrafish embryogenesis, single-cell RNA sequencing, lineage specification, cardiac progenitors, pseudotime analysis

## Abstract

Understanding the development and differentiation of cardiac progenitor cells during the initial stages of embryogenesis is central to a complete understanding of vertebrate heart development. In zebrafish, cardiac specification begins during gastrulation; however, the single-cell transcriptional dynamics of initial cardiac lineage commitment remain not fully defined. In this case, we integrated single-cell RNA sequencing datasets of zebrafish embryos at 4 and 6 h post-fertilisation (hpf) to investigate early cardiac lineage specification. The unsupervised clustering of the integrated dataset identified 12 distinct cell clusters, which made it possible to identify a transcriptionally distinct population of cells characterised by the coordinated expression of transcription factors associated with cardiac development. A further subclustering of the cells expressing cardiac-associated transcription factors showed a significant level of early diversification of the cardiac progenitor group. A projection onto low-dimensional embedding revealed a structured transcriptional organisation of the cardiac subclusters, marked by the differential expression of key cardiac transcription factors, including Gata5, Gata6, Hand2, Nkx2.5, and Tbx5a. A pseudotemporal trajectory analysis uncovered a continuous developmental progression within the cardiac lineage and indicated the gene-specific dynamic regulation and temporal hierarchy of cardiac transcriptional programs. Collectively, these results indicate that zebrafish cardiac progenitors are transcriptionally diverse and acquire cardiac fate through a sustained, continuous regulatory process rather than an abrupt fate transition. This work provides an informative, high-resolution model of early cardiac lineage specification and highlights the power of single-cell transcriptomics for analysing dynamic events in vertebrate embryogenesis.

## Simple Summary

Understanding how the heart starts to develop at early stages of life is essential for uncovering the origins of heart defects. In this study, we explored the development of the initial heart-related cells in zebrafish embryos. Although scientists were already aware of the timing of the emergence of these cells, it was not certain whether they all began in an identical manner or whether they were diverse initially. We suggested that these early heart-related cells are not the same by examining the genetic activity of thousands of individual cells using single-cell RNA sequencing technology. Rather, they exhibit great diversity and structured designs at early developmental stages. We found that the process of becoming a heart cell is a gradual, smooth process rather than a sudden one-time event. This study provides a high detail-resolution map of the earliest heart developmental processes. Society can use this knowledge since it enhances our understanding of normal development, which is the initial step towards the prevention or treatment of heart conditions that begin before birth.

## 1. Introduction

The formation of the vertebrate heart is a highly complex process that begins during early embryogenesis. It requires precise temporal and regional control of gene expressions to specify cardiac progenitor cells and direct their differentiation into functional cardiac tissue. In zebrafish, cardiogenesis begins during gastrulation when mesodermal cells become cardiac-competent and subsequently proceed to form the heart tube [1,2]. The zebrafish has emerged as a powerful model organism for studying early cardiac development due to its external embryogenesis, optical transparency and the highly conserved molecular pathways that govern heart development [1,3].

The transcription factors that control early cardiac specification in zebrafish include highly conserved identity and differentiation regulators belonging to the Gata, Hand, Nkx and Tbx families. Specifically, the initial cardiac mesoderm is specified by Gata5 and Gata6, whereas the subsequent commitment of cardiac progenitors and further remodelling of the heart are driven by Nkx2.5 [4,5,6]. Additionally, Hand2 and Tbx5a govern cardiac differentiation and patterning, ensuring the proper development of the myocardial lineage [7,8]. Importantly, these transcription factors are not exclusively specific to cardiac lineages but are also expressed in other embryonic progenitor populations. Despite the extensive understanding of the functional significance of these transcription factors, previous research has largely relied on bulk expression studies or static marker-based systems, which cannot fully capture the heterogeneous and dynamic nature of early cardiac progenitor populations.

The recent emergence of single-cell RNA sequencing (scRNA-seq) has enriched the study of development biology due to the possibility to characterise states and lineages of cells at an unprecedented level. An effective comparison of various stages of development can be achieved using computational integration frameworks, such as those used in Seurat, with minimal technical variation [9,10]. These methods have revealed previously unknown cellular heterogeneity and sustained developmental stages in different embryonic systems [11]. Furthermore, trajectory inference and pseudotime analysis offer powerful computational tools to reconstruct the dynamic transcriptional programs underlying cell fate specifications from static, snapshot single-cell data [12,13].

Despite these advancements, high-resolution single-cell data regarding the initial steps of zebrafish gastrulation, particularly the periods 4 and 6 h post-fertilisation (hpf), remain limited. While the timing of cardiac progenitor emergence has been extensively characterised using classical developmental biology methods, the gradual determination of cardiac fate at the transcriptional level within individual cells remains poorly understood. Specifically, it remains unclear whether early cardiac progenitors constitute a homogenous population or if transcriptional heterogeneity and incremental acquisition of cell fate are already present at such nascent stages.

In this study, we integrate single-cell transcriptomic data from zebrafish embryos at 4 and 6 hpf to examine the emergence and differentiation of the cardiac lineage during early gastrulation. Here, we use the term ‘cardiac progenitors’ in a broad sense to refer to cells expressing transcription factors associated with early cardiac development, without differentiating between specific sublineages (e.g., myocardial or endocardial progenitors). By utilising unsupervised clustering, lineage-specific markers analysis, targeted subclustering, and pseudotemporal trajectory reconstruction, we suggest that early cardiac progenitors are transcriptionally heterogeneous. Furthermore, our findings indicate that cardiac identity is acquired in a continuous, multi-stage regulatory process rather than a discrete fate transition. These findings offer new insights into the chronology and regulatory framework of early cardiac specification in vivo, suggesting the multifaceted cardiac lineage commitment during the earliest stages of heart development in vertebrates.

## 2. Materials and Methods

### 2.1. RNA Sequencing Data on Single Cells

The analysis was performed using publicly available single-cell RNA-seq data from zebrafish embryos (Gene Expression Omnibus (GEO) accession: GSE112294) [14]. According to the original study, each sample was generated from pooled embryos (approximately 50–100 embryos per sample) at defined developmental stages. However, detailed information regarding the number of biological replicates for each stage is not explicitly provided in the available metadata. This dataset comprises droplet-based scRNA-seq profiles from various embryonic developmental stages, including early gastrulation stages. In the present study, analysis was focused on cells at 4 and 6 hpf to investigate the early specification of cardiac-associated cell populations in zebrafish.

### 2.2. Pre-Processing of Data and Quality Control

Raw gene-cell count matrices were processed using the Seurat R package (v5.4). Low-quality cells were removed based on standard quality control metrics, including gene detection thresholds and total transcript counts. Genes expressed in fewer than the minimum number of cells were excluded to minimise noise. After filtering, a total of 9961 cells were retained for downstream analyses, comprising 4269 from 4 hpf embryos and 5692 cells from 6 hpf embryos. The gene expression counts were then log-normalised, and highly variable features were identified separately for each dataset prior to integrating.

### 2.3. Dimensionality Reduction Data Integration

To facilitate a direct comparison between developmental stages, the 4 hpf and 6 hpf datasets were integrated using the Seurat (v5.4) anchor-based workflow. The common highly variable genes were used as anchors of integration to reduce and minimise technical variation but still maintain biological importance in the difference between the developmental periods. After integration, the expression matrix was scaled and dimensionality reduction using Principal Component Analysis (PCA) was done. The most principal components were then utilised to perform unsupervised clustering and visualisation via Uniform Manifold Approximation and Projection (UMAP).

### 2.4. Clustering and Identification of Cell Populations

The integrated dataset was subjected to unsupervised clustering using a graph-based algorithm implemented in Seurat (v5.4). The clustering resolution was determined through an iterative inspection of cluster separation and biological interpretability, which led to finding 12 transcriptionally distinct cell clusters. Cell populations were identified by evaluating expression of established marker genes and comparing them with known zebrafish developmental markers. The coordinated expression of canonical cardiac transcription factors such as *nkx2.5*, *gata5*, *gata6*, *hand2* and *tbx5a* identified cells expressing transcription factors associated with cardiac development.

### 2.5. Subpopulation of the Heart Line Cells

To investigate transcriptional heterogeneity, a cluster-based subsetting approach was used to select cells in clusters 5, 7, and 10 based on enriched expression of cardiac-associated transcription factors, which were re-analysed individually. The cardiac-associated subset underwent re-normalisation and scaling, followed by PCA and UMAP dimensionality reduction. Unsupervised clustering of these cells revealed several transcriptionally distinct subpopulations within cells expressing cardiac-associated transcription factors, which were subsequently characterised through differential gene expression analysis.

### 2.6. Differential Expression and Marker Gene Analysis

Differential gene expression (DGE) analysis was performed using the Seurat framework, employing Wilcoxon rank-sum test to identify marker genes over-represented in each primary cluster and the cardiac-associated subclusters. Marker gene selection was based on statistical significance (adjusted *p*-value) and specificity of expression within each group. The expression patterns of key cardiac transcription factors and developmental regulators were subsequently visualised using dot plots and feature plots to confirm cluster identities and assess transcriptional heterogeneity.

### 2.7. Pseudotime Analysis

Trajectory inference was performed to reconstruct the developmental progression of the identified cell populations. The integrated single-cell data were used to generate low-dimensional embeddings, which served as the basis for inferring a continuous pseudotemporal ordering. For visualisation purposes, pseudotime values were rescaled to a 0–1 range for gene expression trend analysis, while original pseudotime values were retained for UMAP visualisation. To determine how the cardiac-associated cell population is distributed along the predicted trajectory, pseudotime values were mapped to UMAP embeddings. The dynamics in gene expression across pseudotime, specifically concerning major cardiac transcription factors, were analysed to evaluate the temporal regulation and comparative ranking over cardiac regulatory programs during early embryogenesis.

### 2.8. Statistical Analysis and Data Visualisation

All the data analyses and visualisations were conducted using the R programming environment (v4.4.1), primarily utilising Seurat (v5.4) and ggplot2 (v4.0.1) for figure generation. Beyond the statistical test inherent to the differential gene expression analysis (e.g., the Wilcoxon rank-sum test), no additional statistical tests were performed. All computational analyses were executed using default parameters to ensure reproducibility.

## 3. Results

### 3.1. Combination of 4 and 6 hpf Zebrafish Embryo Single-Cell Transcriptomes

**Figure 1:**
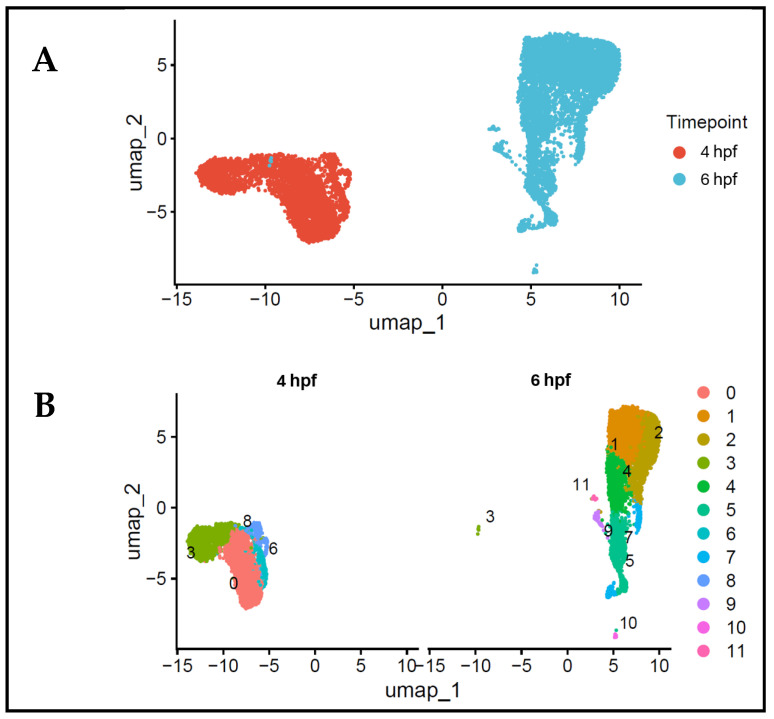
Comprehensive scRNA-seq transcriptomic landscape of zebrafish embryos at 4 and 6 hpf. (**A**) UMAP of the integrated dataset with cells coloured according to the different developmental phase. The transcriptional profile of 4 and 6 hpf cells have their own unique, although partially overlapping, transcriptional signatures that are suggestive of early developmental progression. (**B**) UMAP visualisation colourised by unsupervised clustering, determining 12 transcriptionally distinct cell populations at both timepoints.

To investigate the early changes during zebrafish embryogenesis, we combined single-cell RNA sequencing (scRNA-seq) datasets from 4 and 6 hpf embryos. In line with the existing best practise, we used the known integration frameworks of single-cell transcriptomics analysis [11]. This analysis was performed to reduce technical variation without interfering with biological structure [9,10]. Following quality control and normalisation, a total of 9961 cells were kept and underwent the downstream analysis, consisting of 4269 cells from 4 hpf embryos and 5692 cells from 6 hpf embryos. The datasets were integrated using a common feature space to enable a direct comparison between the developmental stages. A PCA and UMAP dimensionality reduction showed consistent cellular networks and obvious temporal stratification by stage of development (Figure 1A), suggesting a successful integration. The integrated dataset was clustered together through an unsupervised clustering, showing 12 transcriptionally distinct groups of cells (Figure 1B) and providing a basis for subsequent lineage and cardiac-associated cell population identification.

### 3.2. Determination and Characterisation of Cardiac-Associated Cell Populations at 4 and 6 hpf

**Figure 2:**
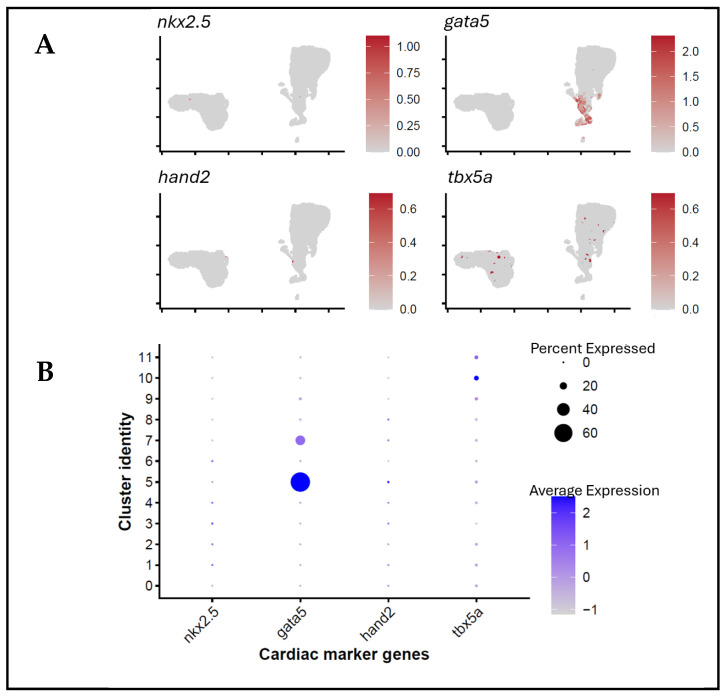

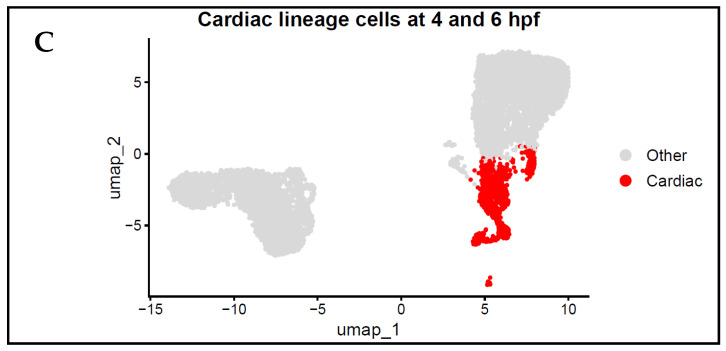
Identification of cardiac-associated cell populations in embryonic zebrafish. (**A**) Dot plot displaying the expression of the canonical marker genes (*nkx2.5*, *gata5*, *gata6*, *hand2*, *tbx5a*, and *lhx1a*) across cardiac-associated and non-cardiac cell groups. Dot size represents the percentage of cells expressing the marker, while colour intensity indicates the average scaled expression level. (**B**) UMAP visualisation of the integrated 4 and 6 hpf dataset, showing 12 transcriptionally distinct clusters. (**C**) UMAP projection highlighting the cardiac-associated cell population (red) relative to non-cardiac cells (grey), demonstrating the emergence of a transcriptionally distinct population by 6 hpf.

The specification of cardiac-associated lineage during gastrulation in zebrafish embryos takes place between 4 and 6 hpf [1,2,3]. To plot these populations at early stages of development, we combined the transcriptomic profiles of the 4 and 6 hpf embryos and used unsupervised clustering methods. This strategy identified 12 transcriptionally different clusters across the developmental stages (Figure 2B). An analysis of canonical cardiac markers (*nkx2.5*, *gata5*, *gata6*, *hand2*, and *tbx5a*) revealed a group of clusters exhibiting a strong and coordinated expression of these genes (Figure 2A). Based on marker enrichment, clusters 5, 7, and 10 were defined as cells expressing cardiac-associated transcription factors and further analysed by subclustering. These cells were predominantly observed at 6 hpf, consistent with the timing of cardiac specification, which aligns with the incremental nature of heart development during gastrulation. The annotation of the lineages onto the UMAP embedding showed that the transcriptionally separated populations revealed a transcriptionally distinct population of cells expressing cardiac-associated transcription factors at 6 hpf (Figure 2C). Collectively, these findings form a transcriptionally distinct group of cells expressing cardiac-associated markers and establish a methodology for the downstream examination of transcriptional changes associated with cardiac-related gene expression during early embryogenesis.

### 3.3. Transcriptional Heterogeneity of Cardiac-Associated Populations Revealed by Subclustering

**Figure 3:**
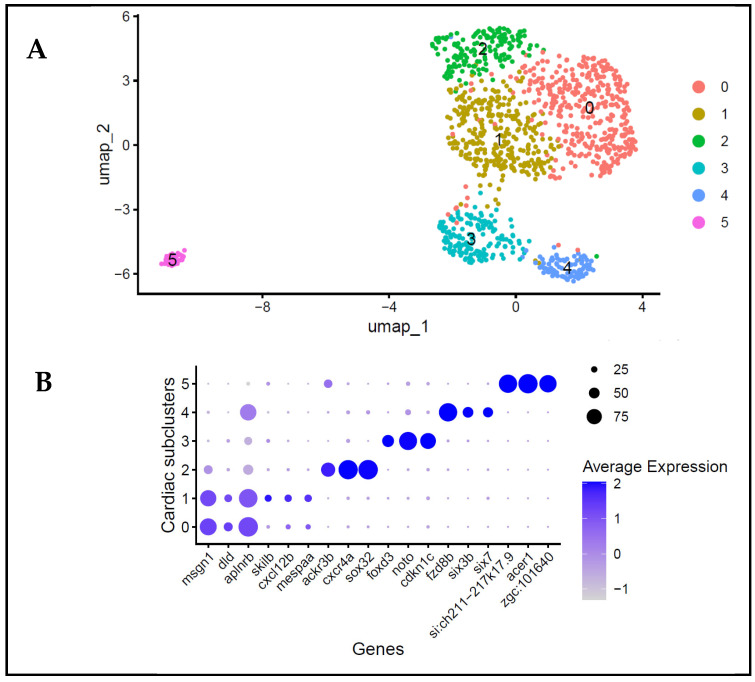
Transcriptional heterogeneity within cardiac-associated cell populations. (**A**) UMAP visualisation of cardiac-associated cell populations following subclustering analysis, revealing various transcriptionally distinct subpopulations within cells expressing cardiac-associated transcription factors. (**B**) Dot plot displaying the expression of selected cardiac-associated transcription factors across each identified subcluster. Dot size represents a percentage of cells expressing the marker, while colour intensity reflects the average scaled expression.

To study heterogeneity, the cells in clusters 5, 7 and 10, defined by an enriched expression of cardiac-associated transcription factors, were reanalysed by subsetting the combined dataset. The unsupervised clustering and dimensionality reduction of these cells showed that the analysed cell population exhibited transcriptional heterogeneity early through the existence of several transcriptionally distinct subpopulations (Figure 3A). The UMAP visualisation indicated that these subclusters were positioned in discrete yet partially overlapping regions within the transcriptional space (Figure 3A), suggesting a progressive lineage commitment rather than an abrupt segregation of fates. These progressive changes align with established models of zebrafish cardiac development during gastrulation, where the progenitor populations are specified in successive stages without being immediately restricted to particular fates [1,2]. The comparative gene expression patterns between the subclusters of the cardiac-associated cell population revealed the presence of differentiated expression profiles, including the enrichment of major cardiac transcription factors and developmental regulators (Figure 3B). The transcriptional heterogeneity in the cell population analysed was demonstrated by the fact that not all of the subclusters had cardiac-associated transcription factors expressed equally. These findings suggest that cells expressing cardiac-associated transcription factors at 6 hpf are transcriptionally diverse and likely represent the early phases of transcriptional changes associated with cardiac-related gene expression, rather than a homogenous progenitor population.

### 3.4. Transcriptional Organisation of Distinct Subpopulations

**Figure 4:**
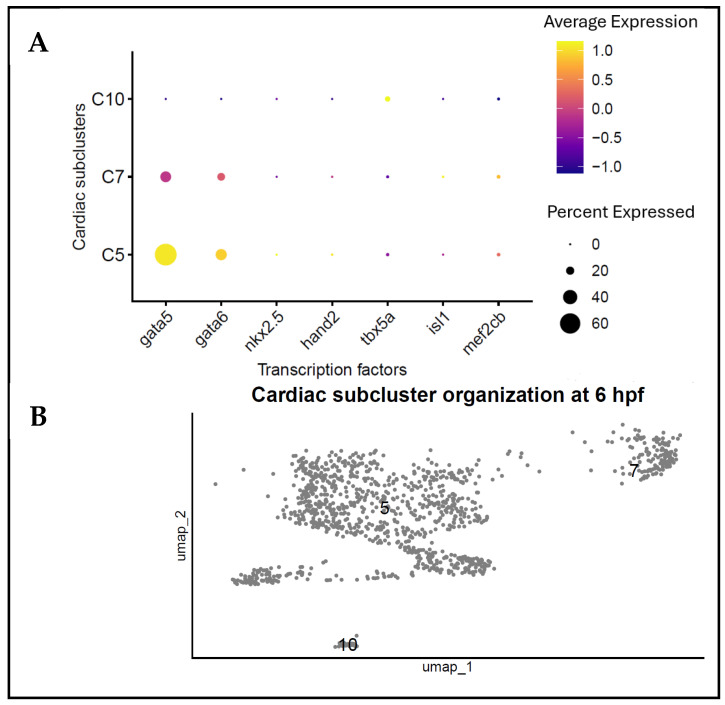

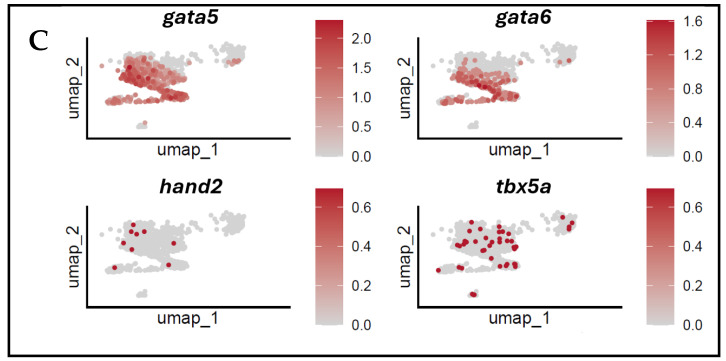
Transcriptional regulation and organisation of transcriptionally distinct subpopulations at 6 hpf. (**A**) Dot plot showing the average expression and percentage of cells which express the essential cardiac transcription factors (*gata5*, *gata6*, *hand2*, and *tbx5a*) across transcriptionally distinct subpopulations. (**B**) UMAP visualisation of transcriptionally distinct subpopulations of the transcriptional space at 6 hpf, with clusters 5, 7 and 10 each taking up a localised area. (**C**) Expression patterns of *gata5*, *gata6*, *hand2*, and *tbx5a* were projected onto UMAP, illustrating subcluster-specific regulatory domains.

To further describe the regulatory properties of the transcriptionally distinct subpopulations, we analysed the expression patterns of the relevant cardiac transcription factors. A dot plot analysis indicated the differential expression of *gata5*, *gata6*, *hand2*, and *tbx5a* across the subclusters within the cardiac-associated cell population, which suggests that there are variations in the regulatory states of the cardiac lineage (Figure 4A). The visualisation of these cells on the UMAP embedding revealed that these subclusters were segregated in transcriptional space, yet formed partially overlapping groups, indicating an underlying structure within the cardiac-associated population (Figure 4B). Notably, clusters 5, 7, and 10 occupied different regions of the transcriptional space, indicating that these subpopulations diverged early. The overlay of transcription factor expression on the UMAP also showed that *gata5* and *gata6* exhibited broadly distributed patterns, whereas *hand2* and *tbx5a* [7,8] showed a more localised distribution (Figure 4C). These areas of localised expression suggest different transcriptional patterns in the subpopulations, although the functional consequences of this heterogeneity are yet to be established. Taken together, these findings indicate that cardiac-associated cells at 6 hpf are not transcriptionally homogeneous but are rather undergoing a coordinated transcriptional organisation with particular regulation programs.

### 3.5. Progressive Transcriptional Regulation of Cardiac-Associated Populations Revealed by Pseudotemporal Dynamics

**Figure 5:**
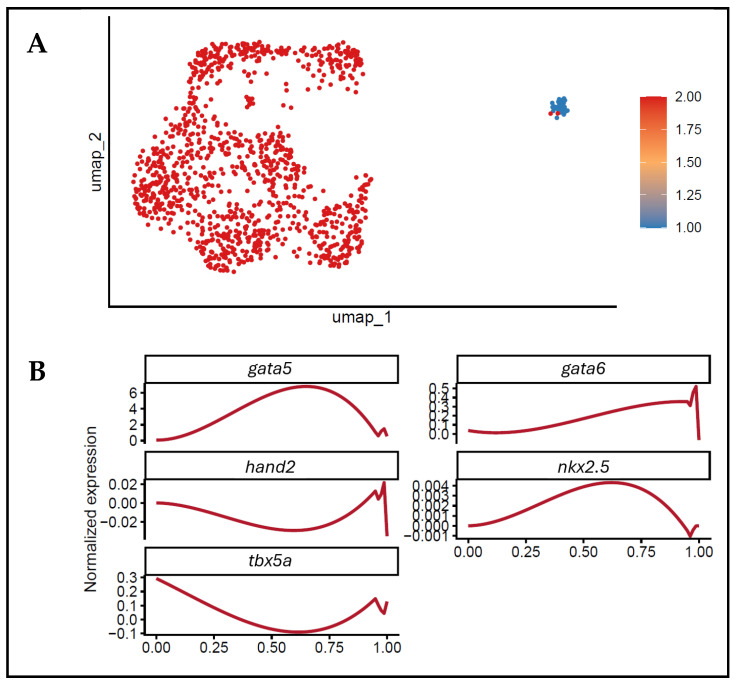
Pseudotemporal transcriptomic dynamics of subclusters within the cardiac-associated cell population at 6 hpf. (**A**) UMAP representation coloured by pseudotime (original scale) of subclusters within the cardiac-associated cell population, suggesting a potential developmental progression across the analysed cell population. (**B**) Gene expression trends plotted against rescaled pseudotime (0–1 range) for key cardiac transcription factors (*gata5*, *gata6*, *hand2*, *nkx2.5* and *tbx5a*), illustrating gene-specific dynamic regulation during early cardiac development.

We carried out a pseudotime analysis on the recognised cell populations to investigate the temporal dynamics of cardiac-associated differentiation at 6 hpf [12]. This methodology enabled the rebuilding of a continuous transcriptional trajectory, which captures dynamic modifications in gene functions in the initial stages of cardiac development [13]. The UMAP visualisation by pseudotime indicated a smooth flow through the analysed cells, but no distinct constant trajectory was clearly resolved (Figure 5A). The cells were spread continuously, which is consistent with progressive transcriptional changes aligned with the early development of the cardiac lineage. It is important to note that the subclusters within the cardiac-associated cell population were positioned at various locations in the pseudotime axis. This distribution proved the premise that the transcriptional heterogeneity of this lineage is not a sharp dichotomy with cell fates, and also not cells in various stages of development. An analysis of gene expression across pseudotime identified the important cardiac transcription factors as being time-controlled (Figure 5B). To make them clearer, the pseudotime values were rescaled to 0 to 1 in the gene expression plots, but the original scale was used in the UMAP visualisation. The early cardiac regulators *gata5* and *gata6* exhibited robust expression during early to intermediate pseudotime, consistent with their established roles in cardiac progenitor specification. In contrast, *nkx2.5* expression peaked at intermediate pseudotime, indicating activation during a transitional commitment stage. *Hand2* and *tbx5a* displayed more stage-specific profiles, with lower expression in early pseudotime and increased levels in later stages, suggesting their roles in subsequent cardiac differentiation and patterning. These findings suggest that cells expressing cardiac-associated transcription factors at 6 hpf undergo coordinated, gene-specific transcriptional changes along a continuous developmental pathway. Consequently, the pseudotime analysis provides insights into the temporal ordering of cardiac regulatory programs and highlights the dynamic activity of the transcription factors during early lineage development.

## 4. Discussion

While the timing of cardiac progenitor development during zebrafish gastrulation has been well established through classical embryological and molecular methods, the initial process of fate commitment at the cellular resolution remains poorly elucidated. The development and diversification of cardiac progenitor cells during early embryogenesis represent a fundamental inquiry in developmental biology. This study provides a comprehensive characterisation of early cardiac-associated lineage specification, transcriptional heterogeneity, and regulatory dynamics during gastrulation. Through the combined single-cell transcriptomic profiles of zebrafish embryos at 4 and 6 hpf, we provide a comprehensive view of these early developmental events. Our analysis suggests that cells expressing cardiac-associated transcription factors exhibit distinct transcriptional profiles and structured subpopulation heterogeneity with continuous transcriptional progression instead of discrete and abrupt transitions between fates.

### 4.1. Early Initiation of Cardiac-Associated Transcriptional Programs

The combination of the 4 and 6 hpf single-cell transcriptomes enabled us to make a direct comparison of the early stages of development and reduce technical variability. The distinct segregation of cells during the developmental stage in the integrated UMAP embedding suggests that considerable transcriptional changes take place even in this limited developmental window. It is worth noting that the unsupervised clustering revealed a discrete cluster of cells with several canonical markers such as *nkx2.5*, *gata5*, *gata6*, *hand2* and *tbx5a*. These data show that cardiac-associated transcriptional programs appear to be initiated early during gastrulation. These observations are consistent with past studies that showed that cardiac progenitor identity is induced during gastrulation by combining signalling and transcriptional regulators, such as the Nodal, BMP and Wnt pathways [1,15,16]. These models are also supported in our single-cell data, where cells expressing cardiac-associated transcription factors exhibit a relatively consistent transcriptional profile that is distinct from other mesodermal lineages at this early stage of development.

### 4.2. Transcriptional Heterogeneity of Early Cardiac-Associated Populations

The subclustering analysis showed that the cells expressing cardiac-associated transcription factors do not form a single, uniform group. Instead, the cells expressing cardiac-associated transcription factors were distributed across multiple transcriptional states, indicating a progressive shift in regulatory programs. This pattern proves a developmental scheme whereby the cardiac-associated groups experience a gradual lineage refinement as opposed to a sudden separation of fates. This early heterogeneity is becoming a characteristic of progenitor populations in different organ systems, the example here being one of them. Recent single-cell studies in zebrafish, mouse and human cells have demonstrated that cardiac progenitors exist in graded transcriptional states influenced by lineage bias, spatial positioning, and developmental timing [17,18,19]. Our findings align with this paradigm, demonstrating that transcriptional diversification within the cardiac-associated lineage is already evident during the early stages of gastrulation.

### 4.3. Transcriptional Organisation and Regulatory Diversity of Cardiac-Associated Subclusters

The analysis of transcription factor expression revealed unique regulatory patterns and an underlying organisation in transcriptional space within the cardiac-associated subclusters. The broad expression of *gata5* and *gata6* across these populations aligns with their established roles in specifying cardiac progenitors and patterning the mesoderm during early development [4,20]. Conversely, the localised distribution of *hand2* and *tbx5a* suggests a possible variation in transcriptional states, but a functional differentiation cannot be inferred based on these data in the cardiac lineage. While chamber-specific patterning and lineage diversification in later developmental stages are known to be spatially structured by transcription factor expression [21,22], our findings at 6 hpf represent an earlier stage of this process. Our results could suggest early regulatory organisation, but the inferences are made based on transcription factor expression patterns, and they do not characterise regulatory networks. The distinct positioning of the subclusters within the transcriptional space further supports the hypothesis that early cardiac precursors are compartmentalised into regulatory domains that may diverge in their developmental trajectories. Although different transcriptional patterns were observed, additional experimental confirmation is needed to determine whether these variations represent functional specialisation.

### 4.4. Continuous Transcriptional Evolution Characterised by Pseudotime

The pseudotime reconstruction of the cells expressing cardiac-associated transcription factors showed a gradual trend in transcriptional dynamics, as opposed to a bifurcating pattern. This observation suggests that the regulation of cardiac progenitors at these early stages is achieved by the progressive transition of regulatory stages. Overlapping gene expression programs characterise this process, as opposed to successive steps of lineage maturation. Such patterns in the distribution of the cardiac-associated subclusters over the pseudotime axis indicate that the heterogeneity observed is due to developmental progression and not a developmentally segregated set of destinies. The dynamic patterns of expression of the major transcription factors across pseudotime also support this model. The early enrichment of *gata5* and *gata6*, which is followed by the intermediate activation of *nkx2.5* and the eventual rise in *hand2* and *tbx5a*, is consistent with temporal hierarchies in cardiac gene regulatory networks [23,24,25]. These results suggest that cardiac-associated population differentiation is regulated by highly coordinated, stage-specific programs that drive a progressive transcriptional change over pseudotime.

### 4.5. Implications for Models of Early Heart Development

Collectively, our findings support a model whereby early cardiac-associated cell populations are characterised by transcriptional organisation into specific regulatory subpopulations, which undergo the successive transcriptional states. This view is opposed to those that put forward early binary fate decisions; rather, it fits with new developmental plasticity and progressive lineage restriction theories. Zebrafish provide a special opportunity to capture such early events due to their external growth and the rapid generation of embryos. The paper fills important gaps in the existing literature since it investigates the earliest phases of gastrulation, and it is complemented by other single-cell atlases that mainly describe the later phases of cardiac morphogenesis and differentiation [14]. It is important to note that the regulatory principles herein identified are probably preserved among vertebrates, as cardiac transcription factor networks have a high level of evolutionary conservation.

### 4.6. Limitations and Future Perspectives

Although the current study offers important information on the dynamics of the early cardiac-associated lineage, it is important to note several limitations. First, our analysis is based on transcriptomic data and does not directly measure chromatin accessibility, signalling activity, or protein-level regulation. Combining these results with single-cell epigenomic or spatial transcriptomic technologies would further enrich our knowledge of the regulation of early cardiac specification. Second, pseudotime effectively describes transcriptional progression, but is a computational proxy, not a measure of chronological developmental time, and should be interpreted with caution. More multimodal data and the extension of this model to later developmental stages will be necessary in future studies to clarify how transcriptional heterogeneity in early development is converted into mature cardiac cell types and functional heart structures.

It is necessary to mention that the findings rely on transcriptomic analysis and do not involve lineage validation in vivo; as such, the identified populations can be seen as putative or transcriptionally defined cell states. Further investigation with lineage tracing and functional validation in vivo will be required in the future to validate the developmental identity and fate of these cells.

## 5. Conclusions

Altogether, this study provides a high-resolution single-cell transcriptomic map of early zebrafish embryo cardiac-associated lineage specification. By integrating the datasets of 4 and 6 hpf embryos, we characterise transcriptionally defined cell populations associated with cardiac development. We also reveal the presence of transcriptional heterogeneity and organisation within the lineage, demonstrating that regulatory progression occurs continuously across pseudotime. These discoveries enhance our understanding of early heart development and provide an important framework for studying the molecular pathways that govern cardiac lineage commitment.

## Data Availability

The single-cell RNA sequencing data used in this paper are publicly available in the Gene Expression Omnibus (GEO) database with accession number GSE112294. All the data generated or analysed during this study are included in this published article. The data processing and analysis was done using custom scripts, which are available from the corresponding author upon reasonable request.
